# Two-Level Evaluation on Sensor Interoperability of Features in Fingerprint Image Segmentation

**DOI:** 10.3390/s120303186

**Published:** 2012-03-07

**Authors:** Gongping Yang, Ying Li, Yilong Yin, Ya-Shuo Li

**Affiliations:** School of Computer Science and Technology, Shandong University, Jinan 250101, Shandong, China; E-Mails: gpyang@sdu.edu.cn (G.Y.); liying200606@163.com (Y.L.); lys.syl@163.com (Y.-S.L.)

**Keywords:** fingerprint segmentation, feature evaluation, sensor interoperability, segmentation error rate, decision tree

## Abstract

Features used in fingerprint segmentation significantly affect the segmentation performance. Various features exhibit different discriminating abilities on fingerprint images derived from different sensors. One feature which has better discriminating ability on images derived from a certain sensor may not adapt to segment images derived from other sensors. This degrades the segmentation performance. This paper empirically analyzes the sensor interoperability problem of segmentation feature, which refers to the feature’s ability to adapt to the raw fingerprints captured by different sensors. To address this issue, this paper presents a two-level feature evaluation method, including the first level feature evaluation based on segmentation error rate and the second level feature evaluation based on decision tree. The proposed method is performed on a number of fingerprint databases which are obtained from various sensors. Experimental results show that the proposed method can effectively evaluate the sensor interoperability of features, and the features with good evaluation results acquire better segmentation accuracies of images originating from different sensors.

## 1. Introduction

Fingerprint segmentation is an important pre-processing step in automatic fingerprint recognition system [1]. A fingerprint image usually consists of two regions: the foreground and the background. The foreground which contains effective ridge information is originated from the contact of a fingertip with the sensor. The noisy area at the borders of the image is called the background. Fingerprint segmentation aims to separate the fingerprint foreground area from the background area. Accurate segmentation is especially important for the reliable extraction of minutiae, and also reduces significantly the time of subsequent processing.

Various fingerprint segmentation methods have been proposed by previous researchers, which can be roughly divided into two types: block-wise methods [2–8] and pixel-wise methods [9–12]. Block-wise methods classify the image blocks into foreground and background based on the extracted block-wise features, and pixel-wise methods classify pixels through the analysis of pixel-wise features. According to whether the label information is used, the fingerprint segmentation methods can also be treated as unsupervised [5–8], supervised [2,3,9,11] and semi-supervised ones [13,14].

Fingerprint images collected by different sensors usually have different characteristics, quality and resolution. However, most fingerprint recognition systems are designed for fingerprints derived from a certain sensor, and when dealing with fingerprints derived from other sensors, the performance of the recognition systems may be significantly affected. Therefore, fingerprint recognition systems encounter a sensor interoperability problem. Sensor interoperability is defined as “the ability of a biometric system to adapt to the raw data obtained from a variety of sensors” [15].

Fingerprint segmentation, a crucial processing step of the fingerprint recognition system, inevitably encounters the sensor interoperability problem. There are mainly two reasons for this [8]. On the one hand, a feature obtained from different sensors may be confused, which results in a block or a pixel being regarded as different categories under views of different sensors. On the other hand, segmentation models trained on one database collected by a certain sensor need to be retrained when dealing with images derived from other sensors.

Much attention has been paid to the sensor interoperability problem of fingerprint segmentation. The works [8,13,16] usually follow two directions: (1) extracting features with interoperability and (2) designing segmentation methods with interoperability. In [16], Ren investigated the feature selection for sensor interoperability and took fingerprint segmentation as a case study. Studies show that features exhibit different sensor interoperability in images derived from various sensors. Variance is found to be an interoperable feature in fingerprint segmentation. In [8], we empirically analyzed the sensor interoperability problem in fingerprint segmentation, and proposed a k-means based segmentation method to address the issue. In [13], Guo proposed a personalized fingerprint segmentation method which learns a special segmentation model for each input fingerprint image and gets over the differences originated from various sensors.

In this paper, we first empirically analyze the sensor interoperability problem of features in fingerprint segmentation. Then a two-level method is proposed to evaluate the sensor interoperability of commonly used features. This method preliminarily evaluates the feature(s) through the first level evaluation based on segmentation error rate, the feature(s) whose segmentation error rate is high and not stable will be eliminated; the remaining candidate feature(s) will participate in a second level evaluation which is based on a decision tree, and the feature or feature set with good sensor interoperability will be selected according to information theory. The effectiveness of the proposed method is validated by experiments performed on a number of fingerprint databases derived from various sensors.

The paper is organized as follows: Section 2 analyzes the sensor inoperability problem of features in fingerprint segmentation through empirical studies. Section 3 proposes our two-level feature evaluation method. Section 4 reports the experimental results. Finally, Section 5 draws conclusions and discusses future work.

## 2. Sensor Interoperability Problem of Feature in Fingerprint Segmentation

In fingerprint segmentation, features are an important topic and discriminating features usually leads to favorable segmentation performance. There is abundant research on segmentation features, which mainly focuses on defining the discriminating features. The commonly used features in fingerprint segmentation include gray-level features [2,3,9,17,18] (such as gray mean, gray variance, contrast, *etc*.), texture features [3,5,7,9,19,20] (such as gradient, coherence, Gabor response, *etc*.), and other features [12,21–23] (such as Harris corner point features, polarimetric feature, number of invalidated minutiae, *etc*.).

Due to the differences between sensing technologies, fingerprint images derived from different sensors usually have different characteristics, resolution, quality and so forth. Therefore, various features have different discriminating abilities on images derived from different sensors. In order to investigate the influence of various sensors on the segmentation feature, we randomly selected fingerprints from a number of open databases and analyzed the feature histograms.

The fingerprints were collected from three open fingerprint databases: FVC2000 [24], FVC2002 [25], and FVC2004 [26]. Each open database contains four sub-databases, where the first three sub-databases are derived from three different types of sensors, and the last sub-database is generated synthetically. The sensors used in the open databases are presented in Table 1. Each database consists of a training set of 80 images and a test set of 800 images.

We randomly select 10 fingerprint images from each real sub-database to construct a database containing 90 images. Each of the 90 images is partitioned into non-overlapping blocks of 8 × 8 pixels, and then all the blocks are manually labeled as the foreground class and the background class. For each block, we extract four features of mean [9], variance [9], contrast [17], and gradient [7]. In the following contents, we will compare four features’ histograms of fingerprints from same sub-database and different sub-databases, respectively.

**Figure 1.**
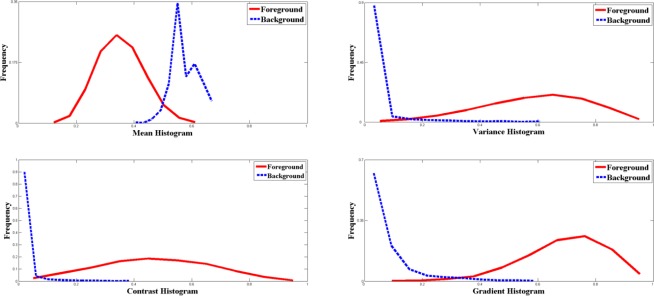
Features’ histograms of 10 fingerprints in FVC2000 DB2.

We first investigate the histograms of fingerprints from same sub-database. A sample result is given in Figure 1 which shows the histograms of mean, variance, contrast, gradient of 10 fingerprints in FVC2000 DB2. We can see that under the view of mean (variance, contrast or gradient), the foreground and background blocks are statistically separable. Actually, the foreground and background blocks of fingerprints from same sub-database can be separated by most segmentation features, and thus for most segmentation features, they have good discriminating abilities and can achieve favorable segmentation performance in images derived from the same sensor.

**Figure 2.**
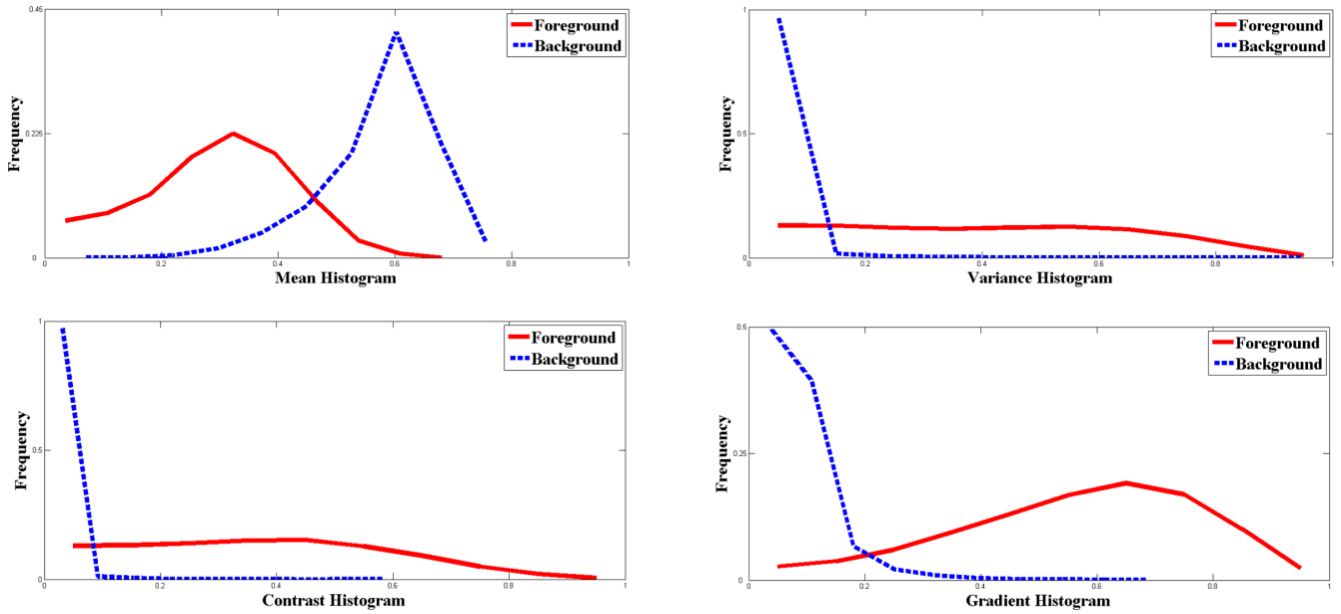
Features’ histograms of fingerprints in three different sub-databases of FVC2000.

Then we compare the histograms of fingerprints from same sensor with that of 30 fingerprints from three different sensors. For example, Figure 2 shows the histograms of fingerprints derived from three sub-databases of FVC2000. Compared with Figure 1, for each feature, we can see that the overlapped area of red line and blue line increases, which shows that the foreground and background blocks are not easy to separate. Therefore, it is not difficult to understand that the discriminating abilities of features on images captured by different sensors reduce and the segmentation performances of these images are not satisfactory. The segmentation features suffer from sensor interoperability problem.

**Figure 3.**
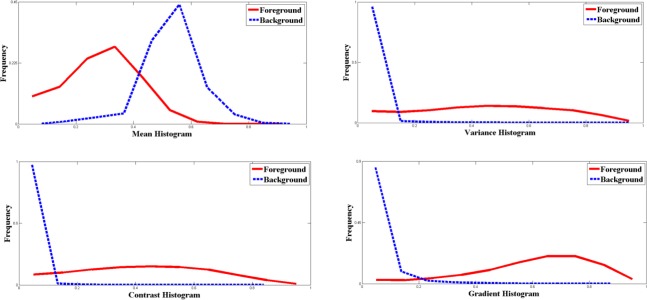
Features’ histograms of fingerprints in 9 different sub-databases of FVC2000, FVC2002 and FVC2004.

Figure 3 provides the features’ histograms of all the 90 fingerprints. In comparison with Figure 2, the overlaps of every feature become more and more complex as the number of different sensors increases. For segmentation features, the more different sensors fingerprints originating from, the more difficult to separate the foreground blocks from the background blocks. This is primarily caused by the sensor interoperability problem.

As the number of different sensors increases, the discriminating abilities of features reduce more or less; however, the degrees of reduction are different for various features. From above three examples, we can see the overlaps of Mean feature become significantly larger as the number of different sensors increases. Therefore, when dealing with fingerprints collected by different sensors, mean feature would not well separates the foreground from background, which results in bad segmentation performance. On the contrary, the change of the gradient histogram is not as obvious. From the gradient histogram in Figure 3, we can see the foreground and background blocks are still statistically separable when fingerprints originated from nine different sensors. Besides, the discriminating abilities of contrast and variance are moderate for fingerprints derived from different sensors.

Feature segmentation, which has an effect on the segmentation performance, is a crucial factor in fingerprint segmentation. Different features exhibit different discriminating abilities on fingerprint images derived from various sensors. Some features can separate well the foreground from the background in images collected by same sensor. However, when dealing with fingerprints collected by different sensors, the discriminating abilities degrade, which results in bad segmentation performance. As mentioned above, a lot of features have been applied to fingerprint segmentation. Which features have good sensor interoperability and adapt to images from various sensors? How to evaluate the sensor interoperability of features and select feature(s) with good sensor interoperability? Can we achieve high segmentation accuracy with selected feature(s) when dealing with fingerprints derived from different sensors? With the above analysis, an effective and robust feature evaluation method should be found to address the sensor interoperability problem of feature in fingerprint segmentation.

## 3. Two-Level Feature Evaluation Method

In order to evaluate the sensor interoperability of features in fingerprint segmentation, we propose a two-level evaluation method, which is composed of two steps. Firstly, all the candidate features participate in the first level evaluation based on segmentation error rate, and the features whose average segmentation error rate is high and not stable will be eliminated; then the remaining candidate features will participate in the second level evaluation which is based on a decision tree, the features will be evaluated according to information gain ratio. Therefore, the two-level evaluation method selects the feature or feature set with good sensor interoperability.

### 3.1. Feature Evaluation Based on Segmentation Error Rate

In the first level evaluation, the mean value and variance of the segmentation error rate are used to evaluate features. The segmentation error rate as a criterion for evaluating a feature is given in [2]. The error rate Err is defined as follows:

(1) $$\text{Err}=\frac{N_{\mathrm{err}}}{N_{\mathrm{total}}}=p(w0|w1)+p(w1|w0)$$

where N_err_ denotes the number of misclassified blocks, N_total_ is the total number of blocks, w0 represents background class, while w1 represents foreground class.

In this step, we make use of a k-means based segmentation method [8] to acquire the segmentation error rate. Since the k-means based segmentation method avoids adjusting thresholds or retraining classifiers for fingerprints derived from various sensors, it ensures sensor interoperability and small time consumption. Firstly, fingerprint images are divided into non-overlapping blocks of the same size, where for each block all the candidate features are extracted. Then for each candidate feature, the k-means based method is performed on each image to get the segmentation result. In the end, the segmentation error rate is acquired by comparing the segmentation result with the manually segmented image.

The mean value of the error rates in images derived from different sensors is a measure to the segmentation performance of every candidate feature. The variance of the error rates reflects the stability of the segmentation performance. The features which have low mean value and variance of segmentation error rates can better adapt to different sensors. Therefore, the mean value and variance of error rate are used to evaluate features finally in the first level evaluation.

**Figure 4.**
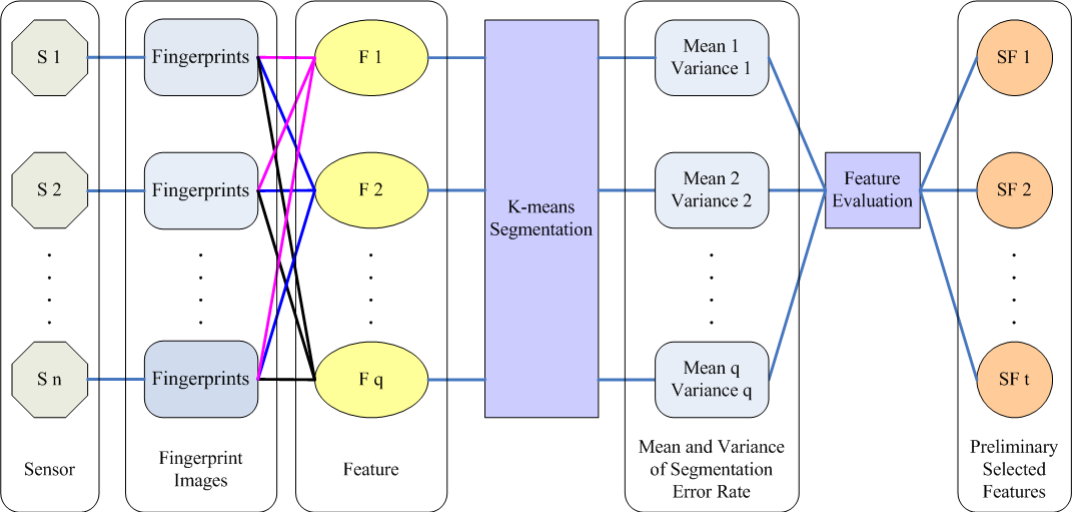
Framework of the first level evaluation.

The framework of the first level feature evaluation is presented in Figure 4. The detail steps of first level evaluation are described as follows:

Step 1: Suppose there are different sensors S_i_ (i = 1 to n). We randomly select p fingerprint images derived from each sensor. Each fingerprint image is partitioned into non-overlapping blocks of same size, and for each block, q candidate features are extracted. F_j_ (j = 1 to q) represent the candidate feature. Then the blocks are manually labeled as two classes: foreground blocks and background blocks.
Step 2: Take the candidate feature F_1_ as example. All the blocks are represented by feature F_1_. The center block of the fingerprint image is initialed as the cluster center of the foreground class, and one border block is regarded as the cluster center of the background one. The k-means algorithm is performed on each image to cluster the blocks into the foreground cluster and the background cluster. The segmentation error rate on each image is acquired by comparing clustering results with manually segmented image.
Step 3: For each candidate feature, the segmentation error rate on one sensor is represented by the average error rate on p images derived from this sensor. After that, the mean value and variance of segmentation error rate on all sensors are computed. Again, we still take the candidate feature F_1_ as example. The segmentation error rate in images derived from sensor S_i_ is named as F_1_E_i_ (i = 1 to n). The mean value F_1_M and variance F_1_V of segmentation error rates are defined as Equations (2) and (3):
  (2) $$F_{1}M=\frac{1}{n}\sum_{i=1}^{n}F_{1}E_{i}$$
  (3) $$F_{1}V=\frac{1}{n}\sum_{i=1}^{n}{\left(F_{1}E_{i}-F_{1}M\right)}^{2}$$
Step 4: For all the candidate features, the mean value and variance of the segmentation error rates are acquired by repeating Step 2 and Step 3. We name the mean value and variance of candidate feature F_j_ as F_j_M and F_j_V (j = 1 to q), respectively.
Step 5: For candidate feature F_j_ (j = 1 to q), if F_j_M < T_m_ and F_j_V < T_v_, F_j_ is selected. T_m_ and T_v_ are empirical thresholds of mean value and variance, which are determined by experiments. In the first level evaluation, we can get the selected feature set {FS_1_, FS_2_ ⋯, FS_t_} (t ≤ q).

### 3.2. Feature Evaluation Based on Decision Tree

**Figure 5.**
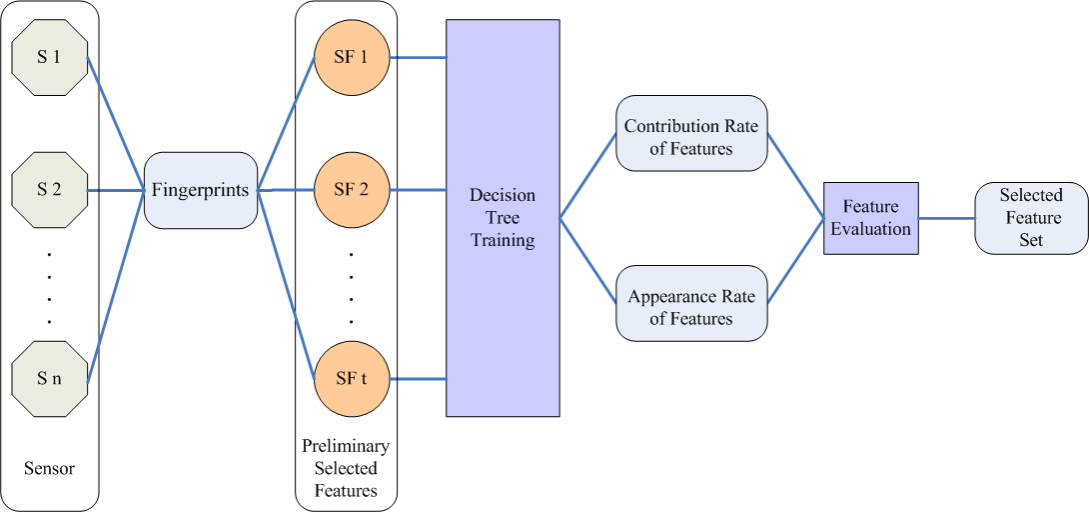
Framework of the second level evaluation.

According to the first level evaluation, the features with high segmentation error rate and instability have been eliminated. The remaining features will participate in the second level feature evaluation. The framework of the second level feature evaluation is shown in Figure 5. In this step, a C4.5 decision tree algorithm [27] is performed to do the selection of the feature set {FS_1_, FS_2_ …, FS_t_}. Decision tree learning is one of the most widely used methods for inductive inference [28]. In the structure of a decision tree, leave nodes represent classifications and branches denote conjunctions of features that lead to those classifications. C4.5 algorithm which chooses attribute by computing gain ratio is one of the-state-of-art decision tree induction algorithms. The gain ratio is defined to be the quotient of information gain and split information [28]:

(4) $$\mathrm{GainRate}(S,A)=\frac{\mathrm{Gain}(S,A)}{\mathrm{SplitInformation}(S,A)}$$

where S and A represent sample set and attribute, respectively.

C4.5 can handle continuous attributes and choose the attributes with largest gain ratio as tree nodes. We regard the features as attributes and evaluate the features using gain ratio.

A number of images captured by sensor Si are randomly selected. Each fingerprint image is partitioned into non-overlapping blocks of same size, and for each block, candidate features FS_1_ to FS_t_ are extracted. In order to perform the following processes, the values of candidate features are normalized into [0, 1] using Min-max normalization. Then the blocks are manually labeled as two classes: foreground blocks and background blocks. For each block, a vector can be acquired as follows:

$$\left({\text{FS}}_{1},{\text{FS}}_{2},{\text{FS}}_{3},\ldots ,{\text{FS}}_{t},\text{Label}\right)$$

All the blocks are used as training samples. A training set is produced by sampling part of blocks without replacement from the training samples. After repeating this k times, k training sets can be acquired. In each training set, a decision tree is trained using C4.5 algorithm. Then two steps will be conducted in each decision tree:

1. **Compute contribution rate of features**. For each feature, the contribution rate denotes the rate of samples classified into the right category through this feature. In a decision tree, leaf nodes represent the class information, by which how many samples are classified into the right category can be obtained. Each path from the tree root to a leaf node corresponds to a conjunction of feature tests. In each decision tree, we firstly compute the rate of samples classified into the right category by each leaf node. Then we retrieve the path from the tree root to every leaf node. Take feature FS_1_ as example. If feature FS_1_ appears in this path, we accumulate the right rate for FS_1_. It is to be noted that if a feature appears more than once in a path, its right rate won’t be accumulated repeatedly. In a decision tree, the sum of the right rates in all paths is defined to be the contribution rate of feature FS_1_. For each candidate feature, the contribution rate CR_j_ (j = 1 to n) is the mean value of contribution rates in k training sets.
2. **Compute appearance rate of features**. For each feature, the appearance rate is defined to represent the quotient of this feature’s total appearance time in all decision trees and the total number of decision trees. It is to be noted that if a feature appears more than once in a decision tree, its appearance time is regarded as one instead of being accumulated repeatedly. We represent appearance rate of candidate feature using AR_j_ (j = 1 to n).

If CR_j_ > Tc and AR_j_ > Ta, the feature FS_j_ is selected. Tc and Ta are empirical thresholds of contribution rate and appearance rate, which are determined experimentally.

According to the two-level evaluation method, we can get the selected feature set {FSs_1_, FSs_2_ …, FSs_m_}, which has good sensor interoperability for images derived from different sensors.

## 4. Experiments

Experiments are conducted using the fingerprint databases listed on Table 1. The experiments involved two parts: we firstly evaluated the sensor interoperability of features on real sub-databases and selected a feature or feature set with good sensor interoperability, then we verified the selected features by applying them to segment fingerprint images. The experiments are conducted under the WEKA platform [29]. We use decision tree (J48) and support vector machine (LIBSVM) implementations in this platform.

### 4.1. Feature Evaluation and Results

In this section, segmentation features are evaluated using the two-level feature evaluation method, and the feature or feature set with good sensor interoperability are selected according to the evaluation results. The candidate features are composed of eight commonly used features, that is, mean(M) [9], variance(V) [9], coherence(Coh) [9], contrast(Con) [17], combination of variance and its gradient(VarG) [18], gradient magnitude(GraM) [7], block clusters degree(CluD) [2], and standard deviation of Gabor features(SDG) [19].

We randomly select 10 fingerprint images from each real sub-database. Each image is partitioned into non-overlapping blocks with the same size of 8 × 8 pixels, and for each block, eight candidate features are extracted. Then the blocks are manually labeled as two classes: foreground blocks and background blocks. For each candidate feature, the first level evaluation based on segmentation error rate is performed. The mean value and variance of segmentation error rate of the candidate features are shown in Table 2. In our experiment, we set Tm = 0.2 and Tv = 0.03, mean, combination of variance and its gradient, block clusters degree, and standard deviation of Gabor features are eliminated from the candidate feature set.

The second level evaluation based on a decision tree is performed on the second level candidate feature set {variance, coherence, contrast, gradient magnitude}. In the second level evaluation, two fingerprint images from each real sub-database are selected. Each image is also partitioned into non-overlapping blocks with the same size of 8 × 8 pixels, and for each block, four candidate features are extracted. The values of these four features are normalized into [0, 1] using Min-max normalization. Then the blocks are manually labeled as two classes, and for each block, a vector can be acquired as follows: {variance, coherence, contrast, gradient magnitude, label}

A training set is produced by sampling 10% blocks without replacement from every image. After repeating this 10 times, 10 training sets can be acquired. In each training set, a decision tree is trained using C4.5 algorithm. After assembling the results in 10 training sets, the contribution rate and appearance rate of every second level candidate feature can be obtained, which are shown in Table 3. We set Tc = 0.1 and Ta = 0.8 in our experiment, the contrast and gradient magnitude are selected finally. The feature evaluation procedure and result are presented in Table 4.

### 4.2. Verification for Selected Features

In this section, we verify the sensor interoperability of the selected features in fingerprint segmentation. Firstly, we compare the selected features with the classic coherence, mean, variance (CMV) [8,9,11] feature combination to show the effectiveness of the evaluation method. Secondly, the segmentation model trained on one database is used to segment fingerprint images in heterogeneous databases and the segmentation performance of the selected features is studied. The segmentation performance is measured by error rate, which is defined in Equation (1).

#### 4.2.1. Comparison to CMV Feature Combination

In the experiment, we compare the selected features with coherence, mean, variance (CMV) feature combination, which is a representative and classic feature combination. For each real sub-database of FVC2000, FVC2002 and FVC2004, 15 images are randomly selected, five images are used to construct a combined database containing 45 images and the remaining 10 images are used for testing. Each fingerprint image is partitioned into non-overlapping blocks of 8 × 8 pixel, and for each block, the selected features and CMV features are extracted, respectively. Then, the blocks are manually labeled as two classes: foreground blocks and background blocks. For each image in combined database, 10% blocks are randomly selected as training samples. The SVM classifiers are trained using selected features and CMV feature combination on the training set, respectively. Then the two classifiers are used to segment other 10 fingerprint images in each real sub-database. The results are shown in Table 5.

Compared with the CMV feature combination, the selected features have lower average segmentation error rates in images derived from different sensors. It is to be noted that the variance of segmentation error rate of the selected features is one third of that of the CMV feature combination. With the above observations we can conclude that the selected features have better segmentation performance for different sensors and the performance is relatively stable. In other words, the selected features exhibit good sensor interoperability.

#### 4.2.2. Heterogeneous Databases Test

In this section, we learn the segmentation model in one sub-database and use it to segment fingerprint images in heterogeneous sub-databases. We randomly select five images from FVC2002DB2 for training and 10 images from each real sub-database for testing. Each fingerprint image is partitioned into non-overlapping blocks of 8 × 8 pixels, and for each block, the selected features are extracted. Then the blocks are manually labeled as two classes: foreground blocks and background blocks. The SVM classifier learnt from FVC2002DB2 is used to segment fingerprint images in all the real sub-databases.

The results are shown in Table 6. The segmentation model which is learnt using the selected features not only has favorable performance in homogenous sub-database but also has lower segmentation error rates in heterogeneous sub-databases. The low mean value and variance of error rate demonstrate the good sensor interoperability of the selected features.

## 5. Conclusions

This work studied the sensor interoperability problem of feature in fingerprint segmentation. We analyzed this problem by investigating the histograms of features in images derived from different sensors. Then a two-level evaluation method is proposed to evaluate features and select a feature or feature set for sensor interoperable fingerprint segmentation. The proposed method implements feature evaluation by the first level evaluation based on segmentation error rate and the second level evaluation based on a decision tree. The first level evaluation eliminates the features with high segmentation error rates and instability, and then the second level evaluation selects the features with largest information gain rate in images derived from different sensors. Experimental results demonstrate that the proposed method can effectively evaluate and select the features with good sensor interoperability, and thus segmentation accuracies of images collected by different sensors are significantly improved with the selected features. The evaluation on sensor interoperability of features also give an impetus to the application of the segmentation algorithms in the internet environment.

Our future works will focus on finding more creative methods to evaluate the sensor interoperability of features and select the features with good sensor interoperability for fingerprint segmentation. Furthermore, we will also conduct research on finding more discriminating features for various sensors.

## Figures and Tables

**Table 1.** FVC fingerprint database sensor list.

**Database Image**	**Sensor type**	**Size**	**Resolution**
FVC2000 DB1	Low-cost Optical Sensor “Secure Desktop Scanner” by KeyTronic	300 × 300	500 dpi
FVC2000 DB2	Low-cost Capacitive Sensor “TouchChip” by ST Microelectronics	256 × 364	500 dpi
FVC2000 DB3	Optical Sensor “DF-90” by Identicator Technology	448 × 478	500 dpi
FVC2002 DB1	Optical Sensor “TouchView II” by Identix	388 × 374	500 dpi
FVC2002 DB2	Optical Sensor “FX2000” by Biometrika	296 × 560	569 dpi
FVC2002 DB3	Capacitive Sensor “100 SC” by Precise Biometrics	300 × 300	500 dpi
FVC2004 DB1	Optical Sensor “V300” by CrossMatch	640 × 480	500 dpi
FVC2004 DB2	Optical Sensor “U.are.U 4000” by Digital Persona	328 × 364	500 dpi
FVC2004 DB3	Thermal sweeping Sensor “FingerChip FCD4B14CB” by Atmel	300 × 480	512 dpi

**Table 2.** The mean value and variance of segmentation error rate.

	**M**	**V**	**Coh**	**Con**	**VarG**	**GraM**	**CluD**	**SDG**

**Mean Value**	0.668972	0.116534	0.135458	0.189248	0.463009	0.067944	0.358899	0.567037
**Variance**	0.070755	0.012847	0.003617	0.022403	0.02774	0.002212	0.033725	0.01576

**Table 3.** The contribution rate and appearance rate of second level candidate features.

	**V**	**Coh**	**Con**	**GraM**

**Contribution Rate**	0.0261	0.0189	0.9847	0.1293
**Appearance Rate**	0.9	0.7	1	0.9

**Table 4.** Feature evaluation procedure and result.

**Procedure**	**Feature Set**

Initial	{M, V, Coh, Con, VarG, GraM, CluD, SDG}
First Level Feature Evaluation	{V, Coh, Con, GraM}
Second Level Feature Evaluation	{Con, GraM}

**Table 5.** Segmentation error rates comparison.

Database	2000 db1	2000 db2	2000 db3	2002 db1	2002 db2	2002 db3	2004 db1	2004 db2	2004 db3	Mean	Variance

**Selected Features**	7.7714%	2.2791%	1.9038%	2.001%	1.8319%	3.0449%	3.729%	4.8062%	3.5961%	**3.44%**	**0.000366**
CMV	7.4939%	1.7442%	3.0312%	2.4753%	2.6303%	3.0041%	0.9637%	4.5021%	10.3399%	4.02%	0.000914

**Table 6.** Segmentation error rates in homogenous and heterogeneous sub-databases.

Database	2000 db1	2000 db2	2000 db3	2002 db1	**2002 db2**	2002 db3	2004 db1	2004 db2	2004 db3	Mean	Variance

**Selected Features**	7.7959%	2.3333%	1.8876%	1.9664%	**1.8361%**	3.0449%	4.2020%	4.8002%	3.3990%	3.4739%	0.000374
